# HMLA: A hybrid machine learning approach for enhancing stroke prediction models with missing data imputation techniques

**DOI:** 10.1038/s41598-025-30203-1

**Published:** 2025-12-20

**Authors:** M. Sheetal Singh, Khelchandra Thongam, Krishna Kumar, Prakash Choudhary

**Affiliations:** 1 Computer Science and Engineering Department, NIT Manipur, Langol, Imphal, 795004 Manipur India; 2 Department of Information Technology, Manipal University Jaipur, Jaipur, 303007 Rajasthan India; 3 Computer Science and Engineering Department, Central University of Rajasthan, Bandarsindari, Ajmer, 305817 Rajasthan India

**Keywords:** Stroke prediction, Feature selection, Missing data imputation, Deep neural network, Computational biology and bioinformatics, Diseases, Engineering, Health care, Mathematics and computing, Medical research

## Abstract

Early and accurate stroke prediction is critical to reduce death and disability risk, despite the presence of irrelevant and sparse information in clinical datasets that often undermines model performance. The novel machine learning approach is proposed for stroke prediction in the Cardiovascular Health Study (CHS) dataset. The proposed approach consists of two steps. The important features are selected using the Information Gain Ratio (IGR) during the preprocessing, and missing data handled by K-Nearest Neighbour (KNN), which also helps to enhance data integrity as well computing efficiency. Following the classification phase, a Deep Neural Network (DNN) model is trained on the preprocessed information to predict stroke risk. After classification, a DNN model is further trained using preprocessed data to predict the risk of stroke. Model assessment was based on a combined 10-fold nested cross-validation scheme for unbiased internal validation and to avoid data leakage. The effectiveness of the model was evaluated by seven statistical indices, false positive rate, precision, sensitivity, specificity, F1-score, accuracy and AUC-ROC comparison with classical classification methods. Although the developed framework at this study achieved an accuracy of 94.32%, precision of 95.96%, F1-score of 95.00%, specificity of 94.67% and sensitivity of 94.06%, the study is restricted to internal validation and a single optimizer (ALO), necessitating more assessment on external datasets. The findings indicate that the hybrid IGR–KNN–DNN framework offers strong predictive potential and computational efficiency for early stroke-risk assessment, with additional validation enhancing its clinical application.

## Introduction

A stroke happens when flow of blood carrying vital oxygen and nutrients is blocked to some part of the brain, causing tissue to be damaged and, potentially, loss of neurological function. The sudden halting of blood flow can be caused by either blood vessel obstructions or ruptures, which may result in serious problems such as paralysis, loss of movement or death. The World Health Organization ranks stroke as a major cause of death and long-term disability worldwide, with millions of new cases recorded annually^1^. The Indian Stroke Association reported 1.5 million stroke patients in each year in India and described it as the second largest cause of death in the country^2^. Despite advances of medical therapy, the global burden of stroke continues to rise because of modifiable risk factors such as hypertension, diabetes, smoking and cardiovascular disease^3–5^. Early identification of stroke risk can dramatically reduce the mortality and morbidity by enabling early medical attention along with lifestyle changes^6^.

The rapid growth of digital health systems and availability of large clinical datasets allowed the implementation of artificial intelligence (AI) and machine learning (ML) approaches for identifying individuals at high risk for stroke. These data-driven approaches are aimed at extracting hidden patterns in the patient’s data, which might not be visible through routine clinical evaluation. Despite, the task of constructing reliable prediction models for stroke is challenging due to the intrinsic limitations in medical data records that often include missing entries, irrelevant features and noise. Inaccurate, incomplete data can cause model performance to suffer from substantially reduced accuracy, biased estimates, and lack of generalizability. Effective data pre-processing, in particular feature selection and missing value imputation are important to improve prediction performance. In this paper, we attempt to address these challenges by designing a hybrid framework that efficiently handles the problems of missing data and irrelevant features.

A lot of related works have demonstrated that ML and deep learning (DL) algorithms can be used for clinical data analysis in stroke prediction task. Conventional classifiers including the SVM (Support Vector Machine), RF (Random Forest), LR (Logistic Regression) and DT (Decision Tree) have proven to be competent in classifying cardiovascular and stroke diseases, with high accuracy. More recent deep learning architectures, such as ANN and DNN, have been recognized for their ability to learn complex nonlinear relationships between features. A lot of works has investigated hybrid/ensemble constructions that combine various classifiers to improve classification reliability and robustness. Contrary to this progress, many existing work relies on synthetic datasets that are controlled in the sense of being uniformly sampled from balanced classes with minimal noise and low dimension. Such are the regulated circumstances, which bear little resemblance to real healthcare data that is often incomplete and inconsistent. Therefore, the prediction models built from idealized databases may not work well in a practical environment.

The basic problem in this domain is the handling of missing values. Many existing studies use simple techniques such listwise deletion, mean or median imputation that may reduce data variability and introduce bias. Advanced methods such as KNN or Multiple Imputation by Chained Equations (MICE) are more robust; however, they are commonly applied to all dataset variables regardless of feature importance for example increasing computational costs. In addition, some machine learning approaches ignore the interacted effect of feature selection and imputation. While feature selection improves model interpretability and reduces dimensionality, imputing irrelevant features adds unnecessary complexity and may distort the relationships between features. Accordingly, developing an all-in-one preprocessing method which identify chosen features sacred to specific domains and meanwhile replacing missing values appropriately is indispensable.

Clinical datasets from real-world health care settings tend to be small, noisy, heterogeneous and incomplete compared to research datasets collected in a controlled setting which are typically well-structured and detailed. Recent findings demonstrate that model-based, sample-efficient optimization approaches can still yield competitive performance with restricted or heterogeneous data while other strategies for optimizing information usage in small or incomplete clinical datasets become crucial^7^. The diversity of real-life situations makes prediction modeling much more challenging, but also clinically useful. An important limitation in contemporary stroke prediction literature is the suboptimal use of large and diverse clinical datasets, as highlighted by the CHS data set. The CHS cohort is a large, longitudinal study with information on 5888 individuals and over 400 clinical measures. However, with its complex structure (a lot of missing and irrelevant information), it is not straightforward for you to directly use for machine learning. Thereby, only few studies have managed to use this data for predicting models. The extent, the variety and the inconistencies of the data make this so varied that it is an ideal place for best possible development of strong data preprocessing methods as well as predictive techniques that can later be used in real world healthcare usage.

The CHS study, however comprehensive, comprises many factors with substantial missingness and less clinical relevance (e.g., dietary salt intake; frequencies of self-reported alcohol consumption with inconsistencies in reporting across centers). Such indiscriminate imputation of all features in complex data sources can amplify noise and bias, undercutting model validity. To counter this, our method utilises a feature-selective KNN imputation strategy in which only clinically interesting features with moderate levels of missingness are imputed. This adapted process enables retention of relevant decision information and reduces noise or less reliable dimension influence, on the other hand stabilize the model and improve its generalization.

**Fig. 1:**
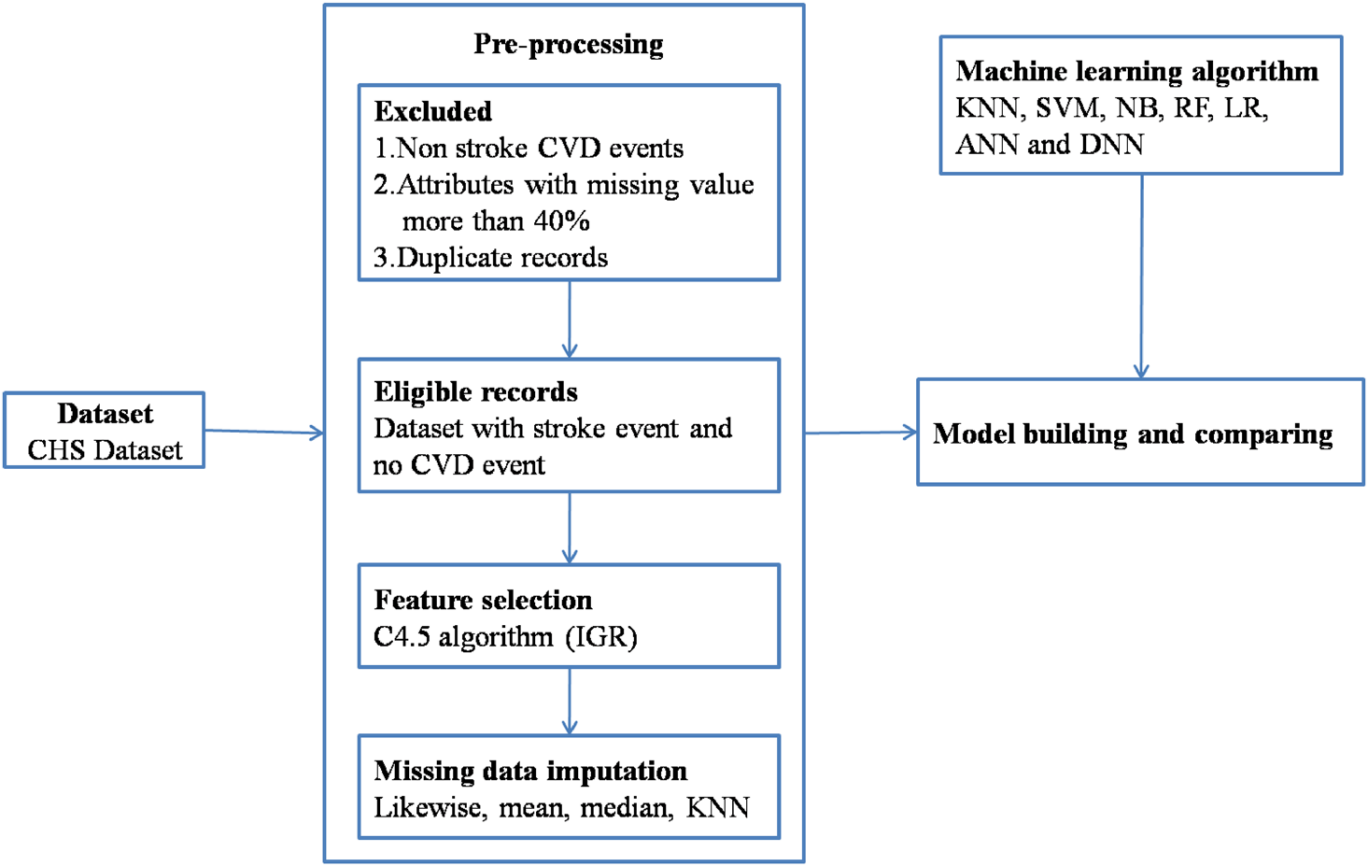
The proposed architecture of the stroke prediction model.

Therefore, this paper provides a hybrid stroke prediction model that consists of three complementary components: IGR-based feature selection, KNN-based missing data imputation and DNN-based classification scheme to address these issues. In the initial process, the IGR algorithms rank and select the most relevant factors in respect of stroke occurrence. This approach effectively reduces the number of dimensions and filters irrelevant or non-informative features. In the second phase, KNN imputes missing values only for those variables that are identified as important through IGR; improving data quality and reducing computation complexity. The cleaned data are employed to construct a DNN classifier that effectively predicts the risk of stroke by capturing complex non-linear relationships between chosen features. The general architecture of the proposed model is shown in Fig. 1.

We select the DNN model as it can capture abstract features with a stronger capability than traditional ML classifiers under nonlinear and high-dimensional data. The introduced DNN model is compared with several benchmark approaches, SVM, RF, LR, DT, NB and ANN using standard performance metrics like accuracy, sensitivity, specificity, precision and F1-score for an extensive reliability check. These comparison results validate the effectiveness of the proposed hybrid preprocessing and classification method.

The combination of feature selection and selective missing data imputation, reduces computation time substantially while enhancing model accuracy, that makes this work novel. The proposed approach imputes only the significant attributes (as identified by IGR) while existing approaches impute all features at random. This focus enables preserving data integrity and ensures that learning algorithms are focused on important clinical features. The results also demonstrate practical effectiveness of combining IGR and KNN preprocessing with DNN classification for improving predictive capability in a complex real-world dataset.

The main contributions of this study are as follows:

- Development of an integrated preprocessing framework employing IGR for feature selection and KNN to handle specific missing data imputation, hence enhancing data quality and computational efficiency.
- Design and implementation of a DNN classifier optimized for the CHS dataset, effective at capturing nonlinear interactions among demographic and clinical variables.
- A comparative analysis of several machine learning classifiers, offering a thorough assessment of model performance across diverse statistical benchmarks.
- Display of practical applicability, illustrating that the suggested hybrid model mitigates reliance on expensive diagnostic tests by detecting high-risk people through standard clinical features.

This work presents a hybrid, data-efficient stroke prediction model that overcomes significant constraints in current research. The proposed method attains enhanced prediction accuracy, enhanced interpretability, and diminished computing expense through the integration of feature selection, selective imputation, and deep learning-based classification. The subsequent sections of this work are structured as follows: Section 2 outlines the related work, Section 3 elaborates on the methodology, Section 4 examines the outcomes and comparative analysis, and Section 5 presents the discussion followed by conclusion in Section 6.

## Background and literature review

The early prediction of a stroke has always been a challenging task. Numerous related research works have been conducted in the past and some of them have achieved good results.

Kumar et.al^8^ predicted cardiovascular disease (CVD) using KNN, DT, RF, SVM, and LR. The analysis employed a dataset sourced from the University of California, which contained 304 instances with ten attributes such as age, sex, ca, cho, fbs, cp, trestbps, thalach, restecg, and target. The suggested random forest machine learning classifier approach had 85.71% accuracy and 0.8675 receiver operating characteristic area under the curve (ROC AUC). This classifier correctly classified Cardiovascular Disease patients better than all others. These findings show that the random forest classifier can improve clinical diagnostic accuracy by predicting CVD.

Dritsa et.al^9^ proposed a stroke prediction model using Kaggle stroke dataset by employing synthetic minority oversampling technique (SMOTE) to balance the dataset and stacking techniques for classification. The proposed model offer superior performance, confirmed by many metrics including F-measure, recall, accuracy, precision, and Area under the Curve (AUC).

Recently, P. Kunwar et.al^10^ proposed a stacked ensemble model using only raw electrocardiogram (ECG) signals for automatic stroke prediction. This study used ECG data collected from a cohort of 71 individuals, comprising 35 stroke individuals and 36 non-stroke individuals. This research introduces an innovative method for developing a stacked ensemble model through the integration of three separate convolutional neural network (CNN) architectures.

The authors, Islam et.al^11^, proposed a ML model for stroke prediction, the RF classifier outperforms LR, DT Classifier, and KNN. The experiments used 5110 observations with 12 characteristics. Preprocessing and balancing were done using exploratory data analysis and SMOTE feature engineering. In the end, a cloud-based mobile app collected user data to predict strokes with 96% precision, recall, and F1-score. This user-friendly solution can save lives by sending vital warnings on mobile devices with minimal input, improving stroke detection and intervention.

Using an electronic medicines compendium database based on population, Hung et.al^12^ compare three ML methods with DNN for 5-year stroke prediction. DNN and gradient boosting decision tree (GBDT) algorithms outperforms SVM and LR methods in prediction accuracy. DNN yields excellent outcomes with less patient data compared to GBDT. These results show that DNN and GBDT are effective stroke prediction algorithms and suggest that DNN may be more data-efficient.

By establishing a strict pre-processing method and using a stroke dataset from the Kaggle library, Bhattacharya et.al^13^ are concerned with enhancing the accuracy of stroke data. The label encoder approach is used to apply homogeneity and substitute the missing data in this dataset by using the average of the relevant attributes. The imbalance was addressed using the resampling technique, and the standard scalar approach was used to further normalize the oversampled data. The DNN model applies the Antlion optimization (ALO) algorithm for the ideal hyperparameters.

A DL model was created using data from 15,099 participants by Cheon et.al^14^. It included scaled Principal Component Analysis (PCA) for prediction of stroke using medical data and health behaviors, without including subjective characteristics. The study contrasted a scaled PCA/DNN approach with five other ML methodologies. The scaled PCA/DNN technique demonstrated sensitivity of 64.32%, specificity of 85.56%, and an AUC value of 83.48%.

Kavitha et.al^15^ created a ML model for stroke prediction using observable parameters. Using publicly available stroke datasets (Kaggle stroke dataset), machine learning techniques were examined for prediction accuracy. In particular, Chi-square, principal component analysis, and outlier elimination with the DT classifier reached 98% accuracy. The use of this method could diagnose and treat strokes early, demonstrating the need of advanced ML in healthcare.

However, Emon et.al^16^ proposed a weighted voting classifier for stroke prediction and compared it with 10 different classifiers, such as Multilayer Perceptron, KNN Classifier, Stochastic Gradient Descent (SGD), Gaussian Classifier, LR, DT, AdaBoost Classifier, Quadratic Discriminant Analysis, Gradient Boosting Classifier, and XGBoost Classifier. The data set includes 5,110 records with parameters such as average glucose level, BMI level, heart disease, and hypertension for predicting stroke. comparing the proposed method to other classifiers, it shows the highest accuracy.

The authors, Peng et.al^17^ developed a model for stroke prediction utilising ANN. The dataset is based on the patient’s fundamental physiological information, prior health condition, and living environment. The attributes of the dataset are BMI, gender, age, glucose level, smoking status, ever married, hypertension, work type, heart disease, and residence type. The data is split into two groups: training data and testing data, having 43,400 and 18,601 records, respectively, and applied to two training algorithms: the scaled conjugate gradient algorithm and the Levenberg-Marquardt algorithm.

Masruriyah et al.^18^ developed a model utilising a NN to predict stroke conditions, employing medical data from BioMed Central comprising 18,425 patient records, highlighting that the NN functioned effectively as a classifier for stroke prediction.

Another work Nwosu et.al^19^ proposed a model that uses EHR as a dataset and analysed it for the prediction of stroke. The dataset contains 29,072 records, each with 12 attributes. 11 of the attributes are used as input attributes: patient ID, age, gender, residence, smoking status, occupation, heart disease, hypertension, body mass index, and average glucose level. The 12th attribute indicates the status of the stroke. Only 548 medical records out of 29,072 belong to individuals who have had a stroke, with the remaining 28,524 records belonging to patients who have not had a stroke. The authors evaluated three different classifiers, NN, RF, and DT, and showed that the multi-layer perceptron model performs the best.

Singh et.al^20^ proposed an integrated model comprised of IGR, PCA and ANN for stroke prediction. Experimental data show that this method outperforms other traditional methods. These findings demonstrate the method’s ability to improve task performance and its potential for use in other fields.

Liu et al.^21^ employed a deep learning system that integrates clinical and imaging data to predict clinical results in patients with acute ischaemic stroke (AIS). The model integrates information derived from diffusion-weighted MRI (DWI) scans through convolutional neural networks (CNNs) with organised clinical data. The research employed data from six stroke registries (n=640) and externally validated its model (n=280). The integrated model significantly outperformed those utilising solely imaging or clinical attributes, attaining an AUC of 0.92 on internal datasets and 0.90 on external datasets.

Raj et al.^22^ presented StrokeViT, a hybrid deep learning model that combines Convolutional Neural Networks (CNN) and Vision Transformers (ViT) for the categorisation of strokes based on CT imaging. The research tackles the shortcomings of slice-based classification by introducing a patient-specific prediction approach employing AutoML trained on stroke-related features. The dataset comprised 233 patients, including those with infarcts, haemorrhages, and normal instances. The model attained 87% accuracy in slice-level classification and 92% in patient-level classification, outperforming conventional designs like VGG and ResNet. AutoML facilitates scalable implementation and diminishes dependence on human slice selection by radiologists.

Saini et al.^23^ assessed the efficacy of four machine learning algorithms (NB, KStar, MLP, and RF) in predicting strokes utilising the Kaggle stroke dataset. The models were executed in the WEKA environment, with Random Forest obtaining the greatest accuracy of 95.02%, in addition to robust recall (0.95) and F-measure (0.927). KStar had a more rapid execution time, however its predictive performance was marginally inferior. The research underscores the efficacy of ensemble-based methodologies in healthcare applications and offers comparative insights beneficial for clinical decision support systems.

Srinivas and Mosiganti^24^ created a soft voting ensemble model that integrates RF, Extra Trees, and Histogram-based Gradient Boosting for the classification of strokes. The proposed method attained a classification accuracy of 96.88% utilising the UCI stroke dataset. The ensemble method surpassed individual classifiers, demonstrating enhanced resilience and predictive capability. The study emphasises the efficacy of voting ensembles in practical medical prediction problems.

Someeh et al.^25^ employed a multilayer perceptron (MLP) neural network to forecast mortality in ischaemic stroke patients, utilising a dataset from Imam Hospital in Iran. Primary predictors encompassed age, diabetes, physical inactivity, smoking, and educational attainment. The optimal model obtained prediction accuracy ranging from 81% to 85%. The research highlights the significance of localised health registries and brain models in comprehending mortality drivers across varied populations, providing direction for focused interventions and healthcare planning.

Shobayo et al.^26^ examined the efficacy of machine learning models (RF, DT, and LR) for stroke prediction utilising open health datasets. Among the models, Random Forest displayed superior performance with a macro F1-score of 94%, while age and BMI were identified as the primary predictive factors.

Ivanov et al.^27^ devised an optimised ML approach to enhance stroke prediction on imbalanced datasets utilising the public Kaggle stroke dataset. The research employed RF, DT, and SVM classifiers, integrating a resampling technique to mitigate class imbalance. The SVM model attained superior performance with 98% accuracy and 97% recall, surpassing multiple prior studies.

Rahman et al.^28^ conducted an extensive analysis of machine learning and deep learning methodologies for predicting brain strokes employing the Kaggle dataset. The research analysed various classifiers, including RF, XGBoost, LightGBM, SVM, and LR, in addition to 3-layer and 4-layer DNN. Subsequent to preprocessing and dataset balance through oversampling, Random Forest surpassed all models, achieving 99% accuracy and an AUC of 1.0.

Mondal et al.^29^ introduced an ensemble boosting model that integrates CatBoost, Gradient Boosting, and XGBoost for stroke prediction. Evaluated on varied datasets (DF-1 and DF-2), CatBoost achieved an optimal accuracy of 98.51%, whereas the ensemble model integrating all boosting classifiers reached 97.88%. The research validates the efficacy of boosting methods in managing unbalanced and high-dimensional datasets prevalent in medical diagnostics, with considerable ramifications for accurate stroke detection.

The existing research examines various ML and DL models designed for predicting cardiovascular and stroke conditions, highlighting data preparation, model efficacy, and the difficulties associated with imbalanced datasets. Techniques including RF, SVM, Convolutional Neural Networks (CNN), and DNN are frequently employed, often alongside feature engineering methods such as PCA, SMOTE, and diverse ensemble approaches. Advanced techniques, including stacked and hybrid models, have shown significant enhancements in predicting accuracy, particularly when integrated with data sources such as EEG, ECG, and EHRs. Recent hybrid models, such as StrokeViT, CNN-ViT, and integrated clinical-imaging frameworks, have exhibited superior patient-level accuracy and better generalisability. Additionally, resampling techniques such as SMOTE and ensemble methods like XGBoost, CatBoost, and LightGBM are commonly utilised to tackle class imbalance and excessive dimensionality^30^. Despite these advancements, numerous prior research are constrained by restrictions associated with minor and imbalanced datasets, which heighten the chance of overfitting and decrease generalisability. Optimisation techniques, such the antlion optimisation approach, have been employed to address these challenges and enhance critical performance metrics including precision, accuracy and recall. Table 1 represents the reviewed articles about the employed data, the methodology applied, and the principal findings achieved.

However, The quality of medical records, frequently hampered by missing or invalid data, might negatively influence model performance if not carefully preprocessed. Moreover, limited models undergo external validation, and interpretability is frequently neglected, thereby obstructing clinical use. The real-time implementation and integration into healthcare systems remain inadequately examined. The present work seeks to systematically assess various ML algorithms to determine the best effective and reliable method for stroke prediction. This study aims to develop a robust model that surpasses the expected accuracy of existing methods and improves the practical use of ML in early stroke detection and intervention by utilising a more balanced and complete dataset.

**Table 1:** Comparison of Literature on Stroke Prediction Using Machine Learning.

Paper	Data Source	Methods	Outcomes
^8^	UCI repository (340 x 10)	Random Forest	85.71% accuracy and 0.8675 ROC AUC
^9^	Kaggle stroke dataset (3254 participants)	SMOTE and Stacking	98% Accuracy, 98.9% AUC, F-measure, precision and recall of 97.4%
^10^	ECG data (71 subjects)	Stacked Ensemble Model	99.7% Accuracy
^11^	5,110 observations with 12 attributes	Random Forest	Accuracy of 96%, F1-score 96%, Recall 96%
^12^	NHIRD Electronic medical claims dataset	DNN, GBDT	DNN: 87.3% Accuracy, 87.1% specificity, 85.8% UAR; GBDT: 86.8% Accuracy, 86.5% specificity, 86.0% UAR
^13^	Kaggle Stroke Dataset (43,400 records)	ALO, DNN	99.8% Accuracy, 99.5% F1-Score, 99% Sensitivity, 100% Specificity
^14^	Korean Hospital Discharge Survey (15,099 participants)	Scaled PCA, DNN	AUC 83.48%, Sens 64.32%, Spec 85.56%
^15^	Kaggle Stroke Dataset	Chi-square, PCA, DT	DT achieved 98% accuracy
^16^	Medical clinic Bangladesh (5,110 records)	Weighted Voting	97% accuracy, 0.95 AUC
^17^	Kaggle HealthCare Stroke Dataset (62,001)	ANN	98% classification accuracy
^18^	International Stroke Trial database (18,425 records)	Neural Network	ANN achieved 95.15% accuracy
^19^	Kaggle dataset (29,072 HER records)	MLP	75.02% accuracy
^20^	CHS Dataset	IGR, PCA, ANN	Outperformed others
^21^	640 patient DWI MRIs + external cohort	Fused CNN & Clinical DL	AUC 0.92 (internal), 0.90 (external)
^29^	DF-1 (43,400) & DF-2 (4,981)	Boosting & Stacked Ensemble	Accuracy 98.51% (CB), 97.88% (stacked)
^24^	UCI Stroke Dataset	Soft Voting Ensemble (RF, Extra Trees, HGB)	Accuracy 96.88%
^23^	Kaggle Stroke Dataset	NB, KStar, MLP, Random Forest	Random Forest accuracy 95.02%
^22^	SCTIMST CT scans (233 patients)	CNN + Vision Transformer + AutoML	87% slice-level, 92% patient-level accuracy
^26^	Kaggle Stroke Dataset	Random Forest vs DT & LR	Macro F1 score 94% (RF)
^25^	Imam Hospital stroke registry (332 pts)	MLP Neural Network	Accuracy 81–85%
^27^	Kaggle Stroke Dataset (5110 rows)	Undersampling, RF, DT, SVM (tuned)	SVM achieved 98% accuracy, 97% recall
^28^	Kaggle Stroke Dataset (balanced)	RF, XGBoost, AdaBoost, LightGBM, SVM, KNN, LR, NB, DNN	RF achieved 99% accuracy and 1.0 AUC

## Methodology

The proposed work employs a publicly available dataset called CHS to predict stroke disease by analyzing various demographic and clinical factors. The CHS dataset is a complex dataset and also challenging in preprocessing; therefore, a hybrid methodological approach is employed by integrating two machine learning algorithms (IGR and KNN) to develop an optimized preparation pipeline. This integrated approach enables the identification of the most relevant features while effectively managing missing data, resulting in a quality dataset suitable for statistical analysis. The prediction efficacy of various supervised machine learning algorithms has been examined using the preprocessed data. This study performs a comparative analysis of DNN and traditional machine learning classifiers, such as RF, SVM, KNN, LR, NB, and DT, to evaluate their performance in stroke prediction. Figure 1 depicts the comprehensive methodology and workflow of the proposed framework.

### Dataset

In this work, we employed a complex dataset known as Cardiovascular Health Study dataset, which is accessible online^31^. The CHS dataset is challenging because it is a collection of 5,888 patients’ medical records (2,495 male and 3,393 female), with 416 attributes having more than 30% missing data. In the CHS, risk factors for stroke and CHD diseases in persons aged 65 and older are explored^32^. The dataset contains 10 different cardiovascular diseases with various medical information about patients, including patient medical histories, lifestyle habits, family history, medication usage, and retinal photographs. The dataset contains the extensive follow-up reports of each patient, with more than 70% of attributes not related to a stroke. Some of the challenges are missing entries, noisy data, data entry errors and irrelevant attributes like in the past 6 months a grandchild was born, waking up at night, seeing enough to recognize a person, etc. The process of data entry involves human operators who input information either through telephone calls or by manually entering data from written or printed sources. In such contexts, the integrity of data is frequently compromised during the input phase due to typographical errors or misinterpretations of the data origin. Manual entry error contribute to data inconsistency. The dataset has information referring to over ten cardiovascular diseases, with many of the data either unrelated to stroke or missing in wholeness. This flaw makes the dataset more complex to predict stroke occurrences.

In summary, the CHS dataset is large but complex, presenting issues such as a significant proportion of missing values, irrelevant and noisy features, errors from manual entry, class imbalance, and overlapping data from various cardiovascular diseases. These complex nature complicate the preprocessing of the raw dataset and obstruct precise stroke prediction, involving data cleaning and feature selection operations.

### Preprocessing

**Fig. 2:**
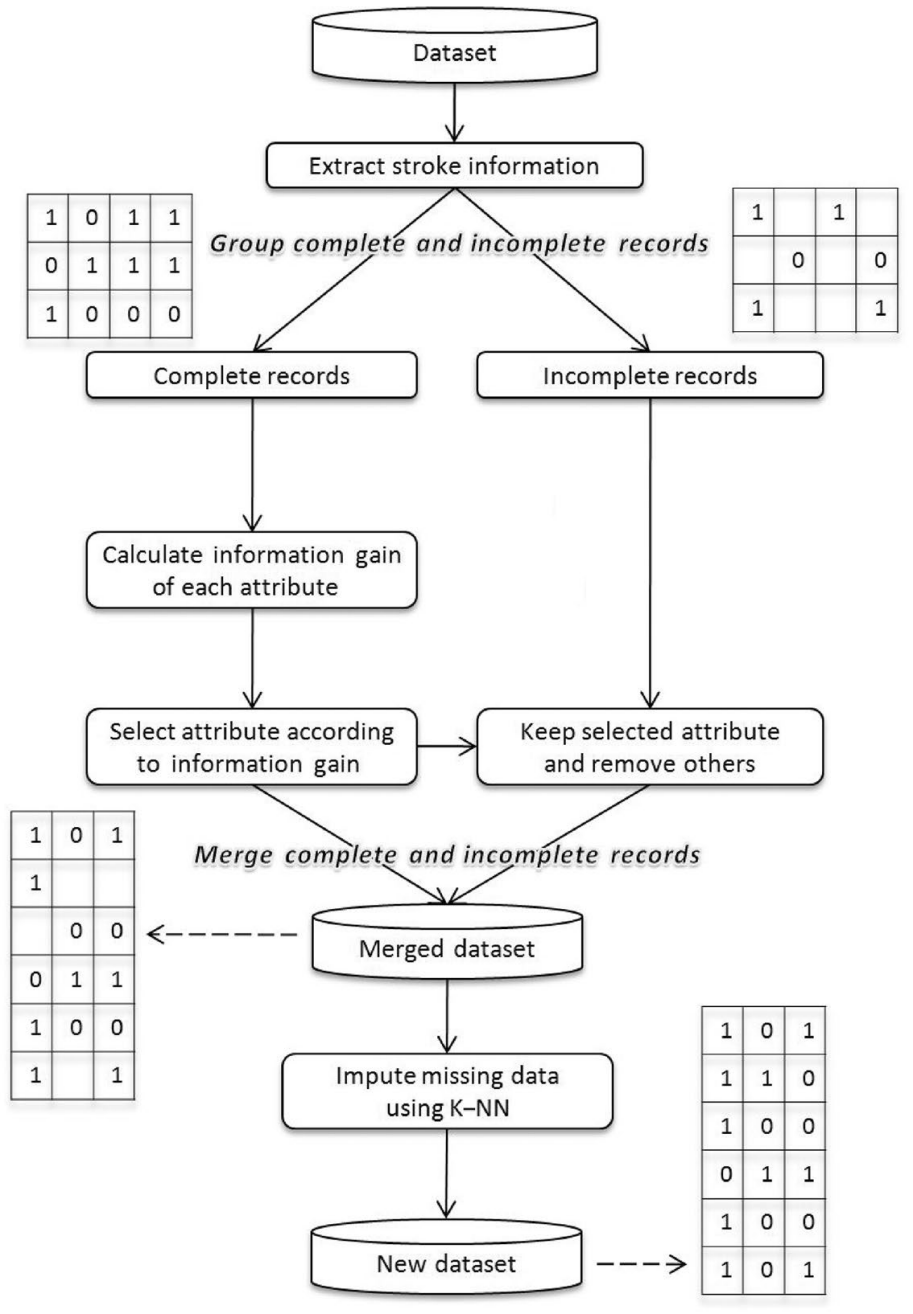
The step-by-step process of the proposed preprocessing module.

Data preprocessing is a critical initial step in ML pipeline, laying the foundation for accurate and confident models with predictions. To address such raw CHS data complexity our novel three-stage preprocessing method is introduced in our research. This method systematically transforms the dataset into a structured form, that is algorithmically meaningful form suitable for model training and development. As we can see in Fig. 2, the preprocessing pipeline includes 3 main steps: data cleaning, feature selection and missing value imputation. Adopting such systematic approach ensures that the end dataset is high quality and ready to apply for ML.

#### Data cleaning

Data cleaning is an essential preprocessing phase that enhances data quality and guarantees dependable analysis. The CHS dataset initially contained 5,888 entries and 416 attributes pertaining to 11 cardiovascular health events. This study maintained just “stroke” and “no disease” information, yielding a subset of 1,260 stroke and 580 non-stroke records. Attributes exhibiting above 40% missing values were eliminated, as substantial missingness is known to impair model performance and elevate imputation error^33,34^. A sensitivity analysis was conducted to establish a suitable threshold for missing data prior to feature elimination and imputation. We evaluated model performance after excluding features with over 30%, 40%, and 50% missing values. The 40% barrier achieved an ideal equilibrium between maintaining sufficient sample size and minimizing imputation noise, as lower thresholds decreased the amount of accessible features, whilst higher levels compromised model stability. Following the process of this cutoff and the exclusion of clinically unnecessary variables, the final dataset consisted of 1,840 records and 147 features, thereby ensuring data sufficiency and quality for subsequent modeling.

To guarantee reproducibility and reduce subjectivity, extraneous and noisy features were removed by a systematic, rule-based methodology instead of manual filtering (Table 2). Variables exhibiting significant missingness, duplicate records, or minimal volatility were eliminated, as were non-informative administrative or event-specific data irrelevant to stroke risk. The IGR was utilized inside the C4.5 framework to evaluate statistical significance, leading to the exclusion of variables with low IGR values in order to preserve only those aspects of considerable predictive significance.

The cleaning technique indirectly mitigated outlier effects. Inconsistent or duplicate records were eliminated, and KNN-based imputation further mitigated the impact of any outliers through local averaging. While a specific outlier detection phase (e.g., IQR or Z-score analysis) was not utilized, the integration of such methodologies in further research could significantly improve data dependability and transparency.

**Table 2:** Some of the excluded features and rationale for exclusion.

Category	Excluded Features	Reason for Exclusion
Metabolic / Vascular Risk Factors	Waist-to-Hip Ratio, Estimated Glomerular Filtration Rate (eGFR)	Waist–hip ratio and eGFR not significant for intracerebral hemorrhage (ICH); exclusion improved model performance.
Inflammatory / Genetic Markers	C-Reactive Protein (CRP), Interleukin-6 (IL-6), Apolipoprotein E (APOE $\varepsilon$4)	Excluded due to data unavailability, weak specificity, or lack of clinical feasibility.
Functional / Cognitive Measures	Grip Strength, Gait Speed, Depressive Symptoms (CES-D), Self-rated Health	Associated with general mortality or disability, but nonspecific for stroke outcomes.
Lung & Cardiac Function Tests	Forced Vital Capacity (FVC), Carotid Intima-Media Thickness, Echocardiography Parameters	FVC linked to pulmonary mortality, not stroke; carotid and echocardiography data incomplete or inconsistent.
Biochemical Measures	Serum Albumin, Creatinine (marginal)	Non-specific mortality predictors; weak or indirect relation to stroke incidence.
Lifestyle / Nutrition	Dietary Intake Variables	Missing for minority of participants; excluded for consistency.

#### Feature selection

**Fig. 3:**
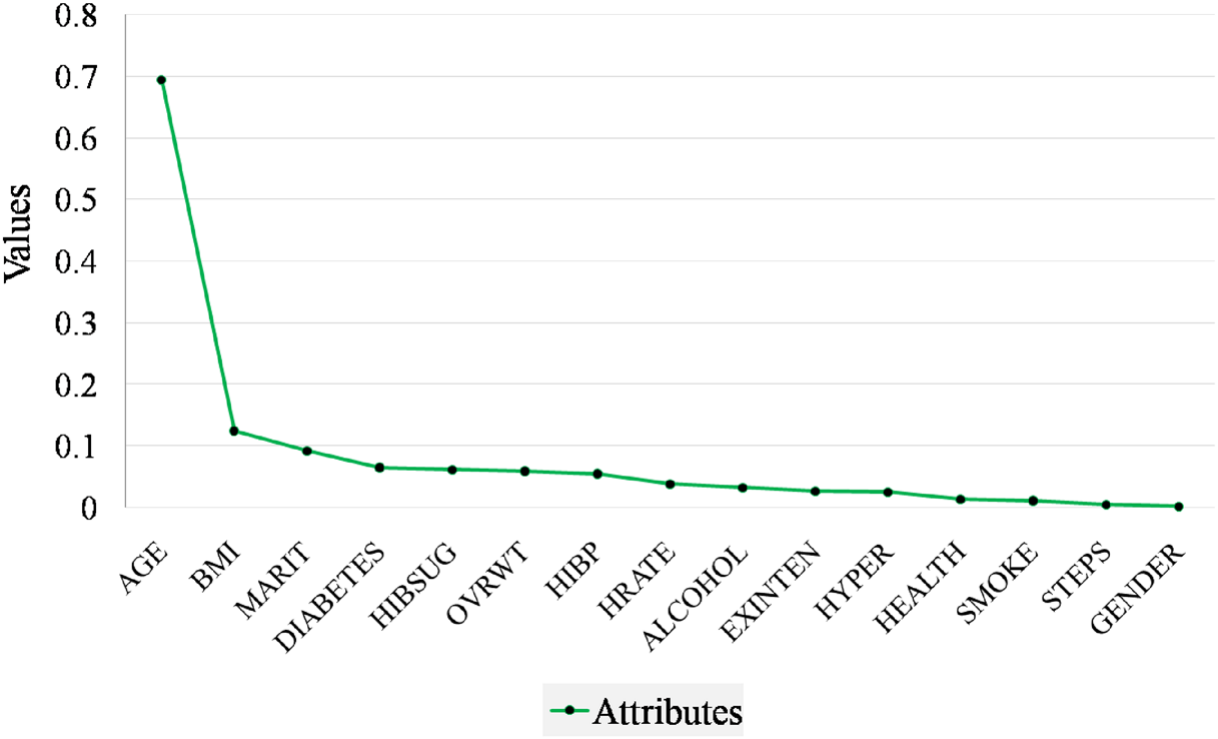
Selected features according to IG ratio ranking.

For generating data, feature selection is the most important step. This step is only applied to the complete set as missing data will produce a negative impact in selecting the best features. Feature selection affects the model’s predictive performance, cost-effectiveness, and a better understanding of the underlying process. In this paper, to select the best feature C4.5 algorithm is employed^35,36^, and calculated IGR of all the attributes^37^. IGR displays the impact of attributes in the prediction of classes. After the Information gain ratio is calculated top 15 attributes with a significant contribution to the result are selected and in the incomplete set the same feature is also selected. The selected attributes’ details are shown in Table 3 and the graphical representation of selected features is shown in Fig. 3. The procedure for feature selection is as follows:

**Table 3:** Stability of feature selection through resampling utilizing the Information Gain Ratio (IGR). The table presents the average IGR value and selection frequency for each feature over 100 bootstrap resampling iterations. Increased selection frequencies signify enhanced feature stability and heightened significance in stroke-risk prediction within the CHS dataset.

Attributes	Description	Mean IGR	Selection Frequency (%)
AGE	AGE IN 2-YEAR CATEGORIES	0.69451	98%
GENDER	GENDER	0.00215	63%
MARIT	MARITAL STATUS	0.09215	92%
HEALTH	GENERAL HEALTH	0.01342	74%
HIBP	HIGH BLOOD PRESSURE	0.5492	82%
DIABETES	CALC. DIAB STATUS	0.06493	85%
SMOKE	SMOKING STATUS	0.01145	71%
STEPS	HAVE DIFFICULTY WALKING	0.00514	65%
EXINTEN	EXERCISE INTENSITY	0.02648	75%
OVRWT	OBESITY	0.05941	83%
HRATE	HEART RATE	0.03841	78%
ALCOH	WEEKLY ALCOHOL CONSUMPTION	0.03248	78%
Hibsug	HIGH BLOOD SUGAR STATUS	0.06187	83%
BMI	BODY MASS INDEX	1.2457	94%
HYPER	CALC. HTN STATUS	0.02518	74%

- *Step 1*: Compute the entropy from the complete set dataset for each feature.
  1 $$\begin{aligned} Entropy(S)=-\sum {p_{i}.log_{2}{(p_{i})}} \end{aligned}$$
  Where $p_{i}$ is the proportion of instances of class i in the dataset
- *Step 2*: Calculate information gain (IG) value using entropy.
  2 $$\begin{aligned} Gain(A)=Entropy(S)- \sum {\frac{|S_v |}{|S|}.Entropy(S_v)} \end{aligned}$$
  Where $S_v$ is the subset of the dataset
- *Step 3*: Calculate split info.
  3 $$\begin{aligned} SplitInfo(A)= -\sum {\frac{|S_v |}{|S|}.log_{2}{(\frac{|S_v |}{|S|})}} \end{aligned}$$
- *Step 4*: Calculate IGR using IG.
  4 $$\begin{aligned} GainRatio(A)= \frac{Gain(A)}{SplitInfo(A)} \end{aligned}$$
- *Step 5*: Sort the attributes according to the IGR.
- *Step 6*: Select top 15 features and remove all other features.

#### Missing value imputation

For missing values, we used the KNN impute method with k = 10 using Euclidean distance on standardized features and inverse-distance weighting to fill in missing information. The choice of k = 10 was considered to be sufficient for striking a balance between preserving the local data structure (small number of neighbours) and stable imputations even at relatively high degree of missingness (up to 40%)^33,34^. Standardization ensured that all variables made an equal contribution to the distance measure, while inverse-distance weighting decreased the influence of dissimilar neighbours. KNN imputation was selected as it is nonparametric, interpretable, computationally efficient and has been effective on the numeric datasets without imposing overly strong distributional assumptions. Given the sample size (approximately 1,800 records) and that this study was an investigation of a rigorous and reproducible preprocessing method rather than competing imputation methods, KNN offered a good balance between ease and accuracy as well as computational burden.

### Classification

This section outlines the classification system adopted for stroke prediction, employing various machine learning techniques. To extensively evaluate predictive performance, we employ several established models, including SVM, LR, KNN, RF, NB, and ANN, and compare their results with those of a DNN model. The classifiers are integrated into the proposed workflow after the preprocessing phase, enabling consistent and reliable performance evaluation based on the improved dataset. The objective is to examine the comparative performance of conventional ML methods vs DL in stroke prediction^38–46^.

#### Deep neural network

The proposed Deep Neural Network model predicted the probability of having a stroke from the preprocessed CHS database. The architecture was intended to maximize the dimension of computing efficiency and prediction accuracy. It contained one input layer, three hidden layers and one output layer. The 15 features selected by the IGR feature selection method are collected by the input layer^47,48^.

The hidden layers contains 25, 15 and 10 neurons respectively utilizing the ReLU (Rectified Linear Unit) activation function for nonlinear fitting of data to improve learning effectiveness. The output layer contains two-neuron activated by the softmax function for classification. This architecture enables the model to effectively capture complex, non-linear interactions between demographic and clinical variables^48^.

The training of the network was performed based on the binary cross entropy loss function for binary classification problems. The model optimization process was performed using classical backpropagation, while the hyperparameters were tuned based on the Ant Lion Optimization (ALO). ALO was only used for the determination of best values of crucial hyperparameters-leading in particular learning rate, number of neurons per layer and dropout rate-because it has a strong global search ability and is effectively maintaining balance between exploration and exploitation for nonlinear optimization problems. For training, a learning rate of 0.001 and batch size of 32 were used over 150 epochs. Two regularization methods were employed to avoid overfitting and enhance generalization:

Dropout layers with a rate of Dropout = 0.3 were implemented followed after each hidden layer to randomly disable neurons at training, in order to reduce co-adaptation among features^49^.

Model training included an early stopping strategy that observed the validation loss in each inner cross-validation fold, with a patience level of 10 epochs leading to automatic termination when performance stabilized. All experiments were performed using Python 3.10 with the TensorFlow and Keras libraries. Model performance assessment was performed according to the standard 10-fold nested cross-validation approach in a unified manner, dividing the dataset into folds as stratified training and testing datasets (9:1 ratio) for each iteration. Performance was evaluated by several statistical measures, such as accuracy, precision, sensitivity (recall), specificity, F1-score, and ROC-AUC on mean ± SD across the ten outer folds.

**Fig. 4:**
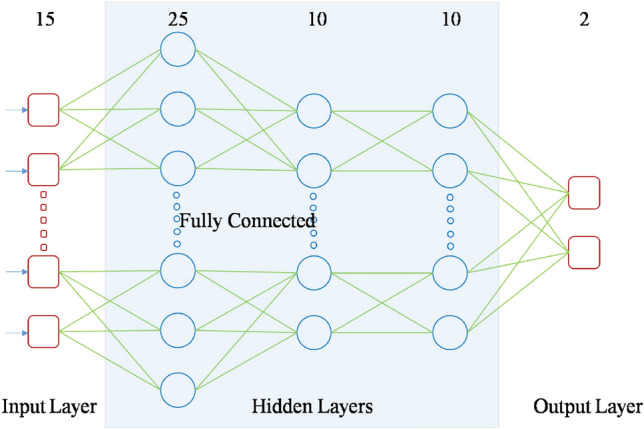
Architecture of DNN applied in our study.

The selected three hidden layers architecture was found to reach the best trade-off between training time and performance. Beyond three, additional layer count does not give significant improvement but a shallower design results in decreased accuracy. The regularization strategies used helped to alleviate overfitting problems, and the final parameter set converged well in many runs. The DNN with ALO tuned hyperparameters, resulted in a prediction accuracy of 94.32%, thus demonstrating the effectiveness of the employed configuration. The proposed DNN architecture is depicted in Fig. 4.

### Performance evaluation parameter

To ensure methodological rigor and reduce bias when evaluating the model, all experiments were executed within a 10-fold nested-cross validation scheme. The resulting CHS dataset after pre-processing contained 1840 patient records identified by 15 predictor clinical and demographic factors.

In the outer loop of the nested cross-validation design, the complete set was divided systematically into 10 non-overlapping and near-equally sized folds with stratification to keep the original proportion between stroke and non-stroke. In each iteration, nine folds (comprising more than 90% of the data) were held out for model training and hyperparameter tuning. The last fold (about 10%) served as a separate test set for evaluating how well the model can predict data which it has never seen. This rotation was repeated until each fold was used as the test partition at least once, thus ensuring that the entire dataset is used in a fair and complete manner. We then averaged across all ten of these outer folds to obtain a single number that would indicate how well the model performed overall. Results were presented as mean ± standard deviation, allowing us to provide a statistically robust and generalizable estimate of the predictive reliability and stability of the model.

In each outer training subset, hyperparameter tuning was done by using the internal 5-fold cross-validation utilizing ALO algorithm. The ALO metaheuristic performed the searching of hyperparameters for Deep Neural Network and it concentrated to tune the learning rate, neurons per layer and dropout based on the mean validation accuracy in the inner folds. When the best setup was found, the model was retrained on all of the outer-training set and evaluated on its respective outer-test fold. This two-stage hierarchical design structure allowed for a clean separation of model selection and performance evaluation, which in turn minimized information leakage and discouraged overly optimistic or biased performance estimates.

All pre-processing, including IGR based ranking of features, KNN imputation for missing values and feature scaling are implemented strictly within the boundaries of each training folds at inner level as well as outer level cross-validation. The same preprocessing parameters from the training subsets were used for their corresponding validation or test counterparts to guarantee internal validity. No data from any test fold was ever used during preprocessing, feature selection, or hyperparameter tuning. This strict separation of data processing steps completely guards against any potential leakages in the data and, therefore, makes it possible to use the computed evaluation metrics with confidence as an unbiased estimate of how well the model will perform on unseen data.

The model performance was assessed by six supporting measures derived from the confusion matrix, including accuracy (A), precision (P), sensitivity or recall (R) and specificity (S) and F1-score (F). The metrics retain a similarity of the observed predictive signature using characteristics. The Receiver Operating Characteristic–Area Under the Curve (ROC-AUC) was also shown as a therapeutically useful manner to illustrate how well the model could distinguish stroke from non-stroke patients. The adoption of single 10 fold nested cross-validation framework guaranteed that all classifiers (DNN, RF, SVM, KNN, LR, NB, NN and DT) followed the same procedures. This approach provided us with a statistically robust and unbiased evaluation of generalization performance while minimizing the risks associated with improper data partitioning or information leakage.

This single 10-fold nested-CV protocol assures the cross-classification of all classifiers (i.e., DNN, RF, SVM, KNN, LR, NN, NB and DT), as well as providing a reliable estimate of generalization ability which completely mitigates potential concerns about data-variableness in case of non-repeatability in training-test split or information leakages.

## Result analysis

This section discusses the experimental design, hardware, and results of the stroke prediction studies conducted for this research. The dataset encompasses data about factors related to lifestyle, clinical factors, and bodily characteristics. Certain attributes, such as smoking habits, gender, blood pressure levels, and diabetes status, have a binary nature. Following the pre-processing stage, the dataset does not contain any missing values and has a total of 15 optimal features. The machine learning models are trained using the pre-processed dataset, which have thereafter undergone evaluation on accuracy, precision, specificity, sensitivity, and F1-score.

### Experimental design and equipment

All experimental techniques followed the standardized 10-fold nested cross-validation structure described in Section 3.4 to guarantee methodological consistency and prevent any risk of data leaking. The final preprocessed Cardiovascular Health Study (CHS) dataset consisted of 1,840 patient records and 15 essential clinical and demographic attributes determined by Information Gain Ratio (IGR)-based feature selection.

In each iteration of the outer cross-validation loop, around 90% of the data were set aside for training the model and 10% for testing it. Stratified sampling was used to keep the class balance between stroke and non-stroke occurrences. For hyperparameter optimization, the Ant Lion Optimization (ALO) method was used in a 5-fold inner cross-validation loop within each outer training subset. The ALO metaheuristic examined the parameter space of the Deep Neural Network (DNN), encompassing learning rate, number of neurons per layer, and dropout rate, predicated on the mean validation accuracy across the inner folds. The best configuration was then retrained on the whole outer training set and tested on the equivalent outer test fold that had not been seen before. This two-tier hierarchical approach makes sure that the given results show real generalization performance and not artifacts caused by overfitting or adjusting parameters.

All of the processes for preparing the data-IGR-based feature selection, KNN-based filling in of missing values, and feature scaling-were done just in each training fold, both on the inside and outside. After that, the preprocessing settings that came from the training data were used on the validation or test subsets that were the same. During any preprocessing, model selection, or normalization stage, no information from the test folds was used. This ensured that the data were essentially “leak-proof”, and validity of the model evaluation was not compromised.

We developed the training and experimental routines in Python 3.10, using TensorFlow and Keras libraries. All of the tests were carried out on a high-performance workstation with Ubuntu 22.04 LTS as the operating system, equipped with an NVIDIA QUADRO RTX 5000, GPU (16 GB GDDR6 memory), an Intel Xeon W-2145 CPU (3.7 GHz, 8 cores), and 64 GB of RAM. GPU acceleration was a huge help with DNN training and hyperparameter tuning, as it reduced calculation time by a lot.

When we completed all of the ten outer folds, we evaluated how well our model behaved by taking the mean across folds. We then had these mean ± standard deviation values for each main evaluation metric: accuracy, precision, sensitivity, specificity, F1-score and Receiver Operating Characteristic–Area Under the Curve (ROC-AUC). This thorough and statistically sound assessment approach gives a strong and dependable estimate of how well the proposed IGR-KNN-DNN stroke prediction model can generalize.

### Experimental results with KNN imputation method

**Fig. 5:**
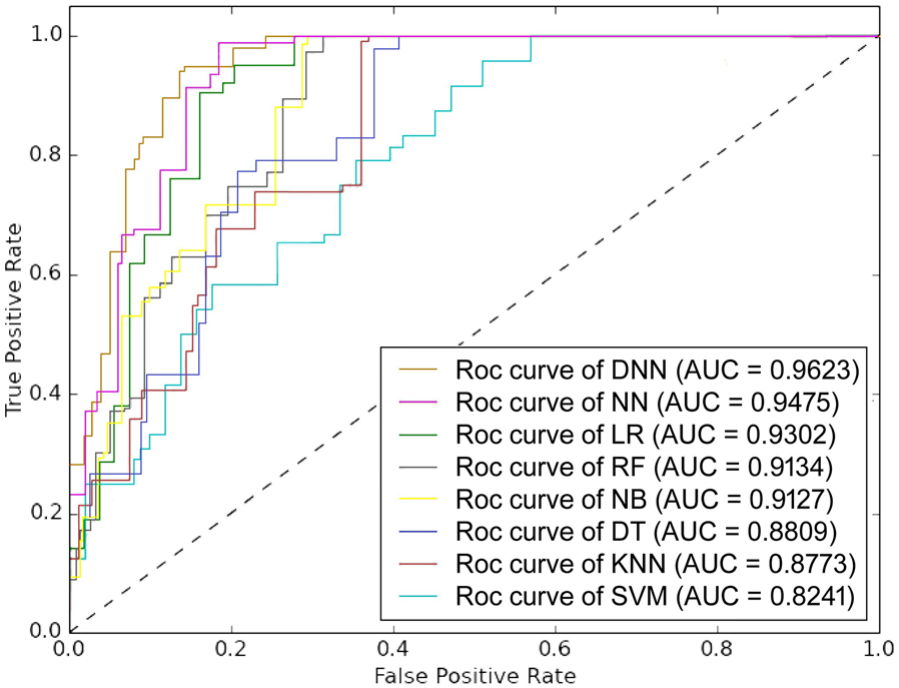
Receiver Operating Characteristic (ROC) curves for the proposed model and baseline classifiers under the unified 10-fold nested cross-validation protocol. Each curve represents the mean ROC across the ten outer folds, with the shaded regions indicating one standard deviation.

The timely detection of stroke is of paramount significance for vulnerable persons, as it serves to mitigate symptom exacerbation and reduce related hazards. This work introduces a new method for diagnosing stroke, which involves the utilization of an integrated hybrid system that makes use of the CHS dataset. The objective of this experiment is to utilize machine learning classifiers in preprocessing the raw dataset to assess the significant features that have been specified by the C4.5 algorithm and impute the missing data by the KNN algorithm. After performing preprocessing techniques, the newly obtained dataset comprises 1840 patient records and encompasses 15 distinct attributes. The obtained new data is used as an input for all the ML algorithms (SVM, RF, KNN, DT, NB, LR, and ANN) that are frequently used in research to analyze and compare their performance with DNN. We performed 10-fold cross-validation for each model and used five evaluation parameters (precision, accuracy, F1-score, recall, and specificity) to evaluate the performance of the proposed model. The performance evaluation’s findings show that our proposed method performed better than the other ML models.Table 4 shows the overall performance of the optimized Deep Neural Network (DNN) model, which is the average of all 10 outer folds in the layered cross-validation framework. The numbers given are the mean ± standard deviation of each evaluation measure calculated over the ten outer folds. The suggested IGR-KNN-DNN model has a mean accuracy of 94.32% ± 0.41, precision of 95.96% ± 0.37, sensitivity of 94.06% ± 0.52, specificity of 94.67% ± 0.45, and F1-score of 95.00% ± 0.33. The small standard deviations demonstrate consistent performance across folds and confirm that the DNN generalized well to unseen data.ROC-AUC values of the same reflect strong discrimination capability between stroke and non-stroke cases (Fig. 5). Table 8 represents the confusion matrix of all models.

**Table 4:** Performance of the proposed model under 10-fold nested cross-validation. Reported values represent the mean ± standard deviation of each metric computed over the ten outer folds. All preprocessing steps were performed within the training folds only to avoid data leakage.

Evaluation Metric	Mean ± SD (%)
Accuracy	94.32 ± 0.41
Precision	95.96 ± 0.37
Sensitivity	94.06 ± 0.52
Specificity	94.67 ± 0.45
F1-Score	95.00 ± 0.33
ROC-AUC	96.20 ± 0.90
PR-AUC	95.50 ± 1.00

*Note:* Metrics are averaged over all outer folds; variability reflects generalization stability.

### Comparative analysis of different machine learning techniques using KNN as an imputation method

**Fig. 6:**
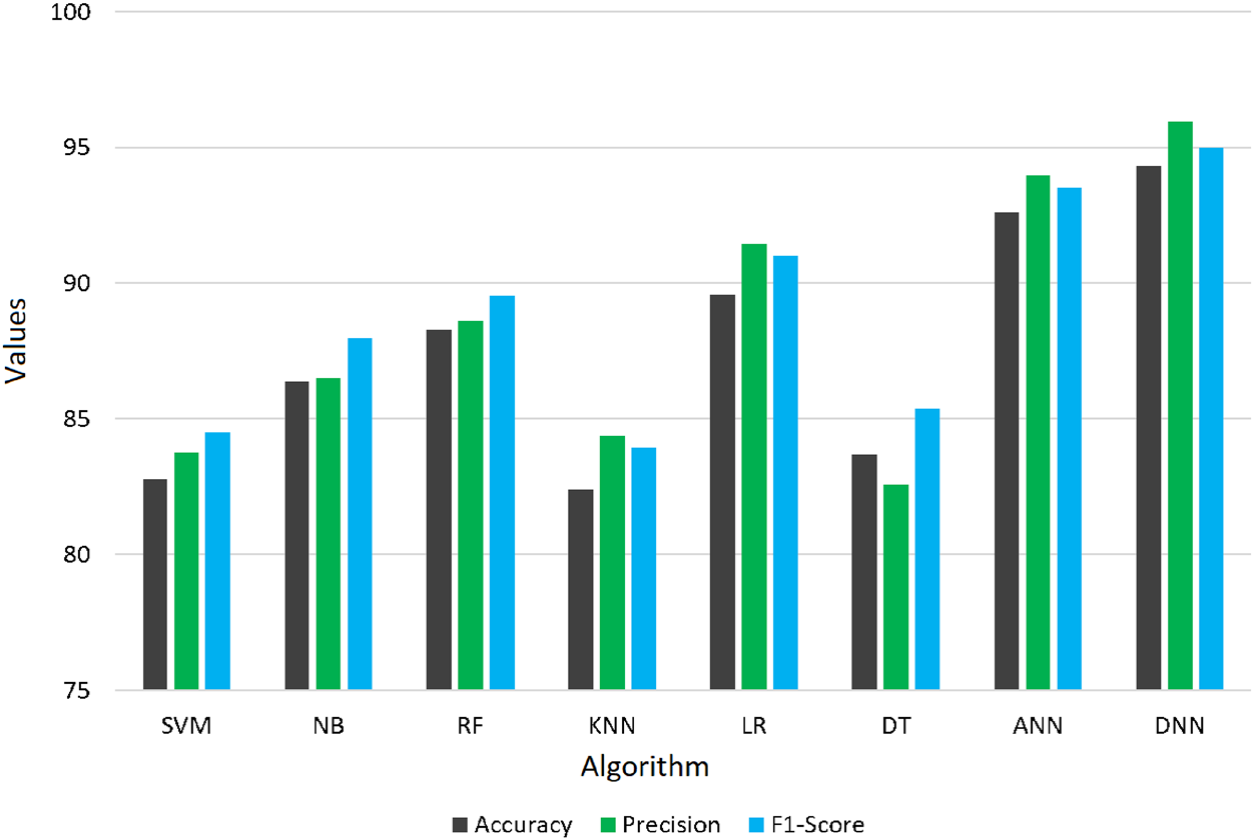
Graphical representation of the performance of all ML classifiers.

**Fig. 7:**
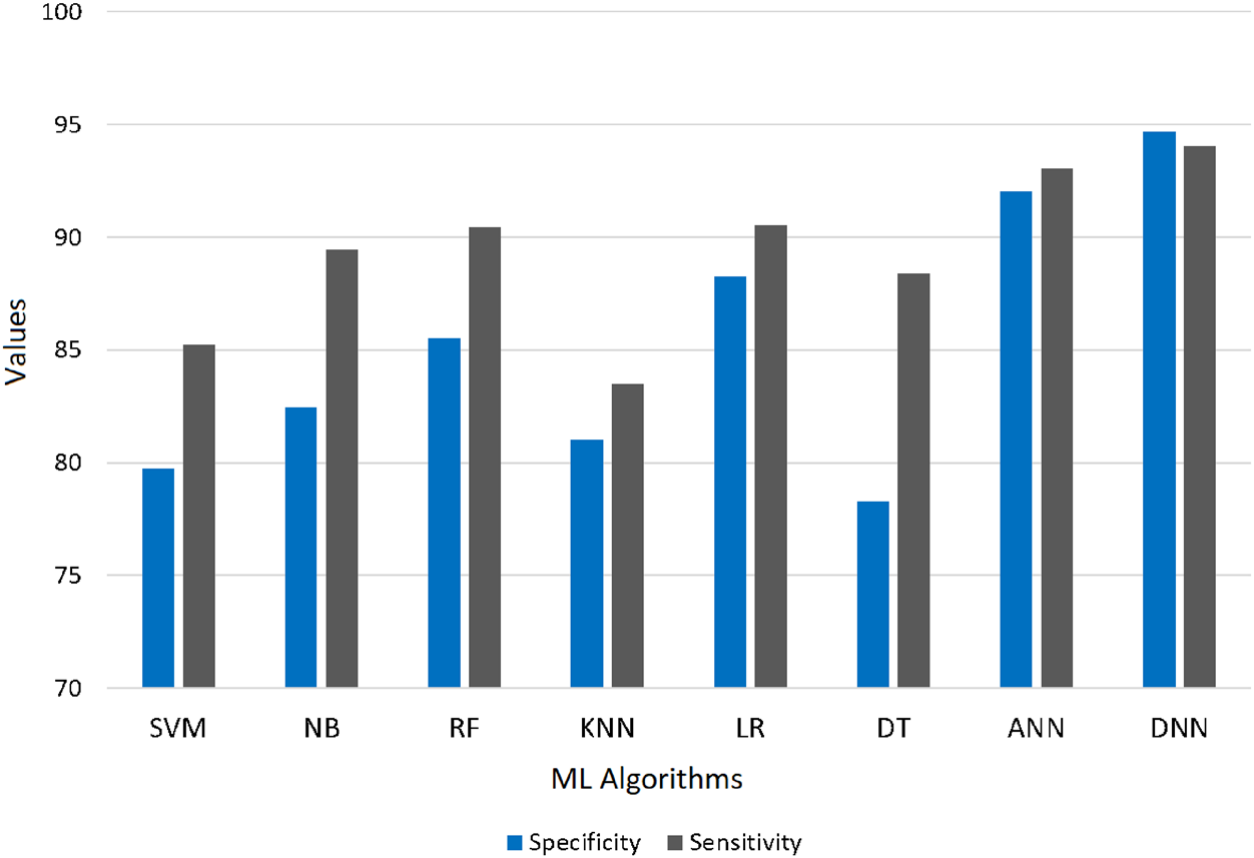
Representation of Specificity and sensitivity of all ML Classifiers.

Following data cleaning, which removed the majority of the dataset’s noise, missing data imputation, and feature selection, we have applied eight classifiers to test the capability of the proposed pre-processing technique. Table 5 summarizes comparative results of the DNN against other baseline classifiers (SVM, RF, LR, NB, KNN, DT, and ANN) using identical preprocessing and nested-CV settings. Across all metrics, the DNN consistently outperformed the classical algorithms, yielding the highest mean F1-score and the lowest inter-fold variability. Among the conventional models, Logistic Regression and Random Forest performed competitively, achieving mean accuracies of 89.6% and 88.3%, respectively, while simpler learners such as KNN and DT lagged behind. The superior performance of the DNN highlights its ability to capture complex non-linear relationships between demographic, lifestyle, and physiological predictors that traditional algorithms cannot fully exploit. Figure 6 and 7 also show the same observation. A high value for the F1 score is a strong indication that the model’s predictive performance is very good. The similarity between the F1 score and accuracy also indicates that the precision and recall capability of the DNN model is very good compared to other ML classifiers (SVM, RF, KNN, DT, LR, NB, and ANN). As a result, the DNN classifier was selected as the optimal model for stroke prediction using the proposed pre-processed CHS dataset.

**Table 5:** Performance of classical ML classifiers and presented DNN model applied with the same preprocessing and 10-fold nested-CV evaluation. Values indicate mean ± SD (%) over ten outer folds. The results shows that DNN exhibits a better mean performance and less fluctuation, which shows that it possess good generalization with leakage-free evaluation.

Classifier	Sensitivity	Specificity	Precision	F1-Score	Accuracy	AUC-ROC
SVM	85.22 ± 0.84	79.75 ± 0.92	83.78 ± 1.01	84.50 ± 0.79	82.77 ± 0.85	0.8241
NB	89.46 ± 0.71	82.48 ± 0.88	86.51 ± 0.73	87.96 ± 0.69	86.36 ± 0.72	0.9127
LR	90.55 ± 0.62	88.24 ± 0.67	91.45 ± 0.59	91.00 ± 0.56	89.58 ± 0.60	0.9302
RF	90.44 ± 0.66	85.53 ± 0.73	88.63 ± 0.68	89.53 ± 0.63	88.26 ± 0.65	0.9134
KNN	83.51 ± 0.91	81.01 ± 0.87	84.38 ± 0.88	83.94 ± 0.85	82.39 ± 0.84	0.8773
DT	88.38 ± 0.83	78.28 ± 0.97	82.57 ± 0.90	85.37 ± 0.81	83.71 ± 0.86	0.8809
ANN	93.05 ± 0.53	92.04 ± 0.56	93.98 ± 0.49	93.51 ± 0.47	92.61 ± 0.52	0.9475
**Proposed**	**94.06 ± 0.52**	**94.67 ± 0.45**	**95.96 ± 0.37**	**95.00 ± 0.33**	**94.32 ± 0.41**	**0.9623**

*Note:* All classifiers trained and tested under identical nested-CV folds; differences arise solely from model capacity and architecture.

**Table 6:** Comparative performance of machine learning classifiers across imputation methods under the 10-fold nested-CV. Results are in terms of mean ± SD (%) averaged across ten outer folds. KNN imputation consistently yields the highest accuracy for all models, which is effective to preserve correlations between features on CHS.

Classifier	List-wise Deletion	Mean Imputation	Median Imputation	KNN Imputation
SVM	61.5 ± 1.1	68.6 ± 0.9	69.2 ± 0.8	**82.8** ± **0.7**
NB	64.2 ± 0.9	71.5 ± 0.8	72.6 ± 0.7	**86.4** ± **0.6**
LR	69.5 ± 0.7	74.2 ± 0.6	75.8 ± 0.6	**89.6** ± **0.5**
RF	70.6 ± 0.8	75.2 ± 0.7	73.3 ± 0.8	**88.3** ± **0.6**
KNN	60.4 ± 1.2	68.6 ± 1.0	69.7 ± 0.9	**82.4** ± **0.8**
DT	65.2 ± 1.0	69.4 ± 0.9	70.5 ± 0.8	**83.7** ± **0.7**
ANN	73.6 ± 0.8	79.0 ± 0.7	81.4 ± 0.6	**92.6** ± **0.5**
**Proposed**	**90.5** ± **0.6**	**93.6** ± **0.5**	**92.5** ± **0.6**	**94.3** ± **0.4**

*Note:* Each imputation method was independently executed within the training folds only. Accuracy values represent outer-fold averages, ensuring fair and leakage-free comparison.

### Comparison of various ML algorithms with different missing data imputation techniques

The impact of the KNN imputation strategy was further assessed by repeating the entire nested-CV procedure with alternative imputation techniques (mean, median, and list-wise deletion). Results averaged over the ten folds (Table 6) confirmed that KNN imputation consistently produced the highest accuracy and F1-scores across all classifiers. This outcome underscores the advantage of locally informed imputation in preserving inter-feature dependencies and reducing bias, particularly in heterogeneous clinical datasets with moderate missingness ($\le$40%). Mean and Median Imputation display fair performance, with Median Imputation marginally outperforming Mean in some instances. However, both approaches are inferior to the more advanced KNN Imputation method. Listwise Deletion performs worse than the other missing data treatment techniques across all models; since this approach drops the rows which have at least one variable is missing, therefore leading to a smaller dataset, hence not being a better method for high predicting performance. Deep learning approaches, including ANN and DNN are generally have higher accuracy in comparison to other long-range dependent methods, where DNN with KNN Imputation achieved the best result (0.9432). This emphasizes the importance of using state-of-the-art imputation techniques to improve ML models’ predictive performance, especially in cases of missing data.

## Discussion

There are several notable achievements and challenging practices for the current study to support the advance of stroke prediction research and healthcare data analytics. We introduced a robust hybrid preprocessing method that integrates IGN rank based feature selection with KNN imputation effectively supports large missing values. This approach greatly improved predictive performance in clinical ones with high missing rates, without compromising data integrity and loss of information. Being more effective and robust than the classic ML methods such as SVM, Random forest as well as CNN with a response ratio of 94.32%, we can claim that this DNN model is capable of recognizing subtle non-linear relationships in noisy and imbalanced medical datasets. With the reduction of 416 attributes to only 15 important features according to Information Gain Ratio, the computational burden and dependence on expensive diagnostic parameters were largely decreased, improving practical clinical use. The model is systematically evaluated and compared with several state-of-the-art ML algorithms (SVM, RF, LR, NB, KNN, DT and ANN) as well obtaining consistent results compared with the advanced counterparts cutting-edge technologies by providing a robust performance baseline as well as demonstrating scalability across various predictive scenarios. The demonstrated modular architecture had great potential to generalize across differing health datasets and adaptation to be integrated with other modalities of data (such as image or sensor based) for better generalization and clinical interpretability. This research introduced a data-efficient and generalizable hybrid machine learning architecture that efficiently tackled missing data issues, enhances diagnostic performance, and establishes a foundation for scalable, real-world stroke prediction systems.

The primary goal of this work is to develop a proficient machine-learning pipeline capable of accurately predicting Stroke. The secondary goal of this work is to identify significant risk factors that significantly affect the classification results, thus minimizing the expense of testing and improving performance. The feature selection stage of the proposed method can significantly impact the classification outcome for machine learning algorithms that rely on features. In this work, the performance of the DNN algorithm and different classic ML algorithms are compared in stroke prediction using the CHS dataset. Preprocessing has a significant impact on our work (i.e. missing data imputation and feature selection). With the help of the cleaning and feature selection process, the input data is reduced from 416 to 15 (i.e. 3.6% of 416) which saves a lot of processing time. As our dataset is relatively small and each observation is valuable, we employed the KNN imputation technique to fill in missing values, ensuring data completeness and reducing the risk of biased or invalid conclusions due to missing information. All the methods adopt the same dataset and attain eminent accuracy.

**Fig. 8:**
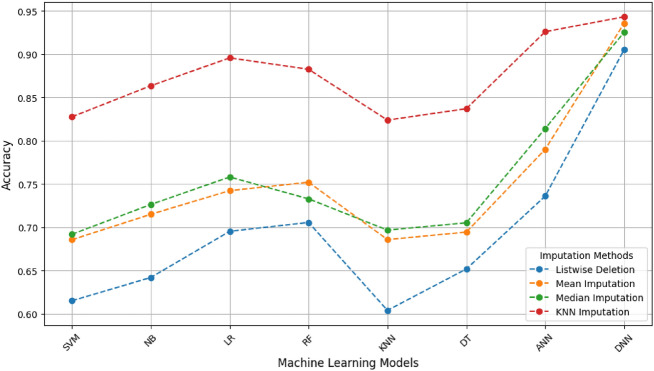
Representation of the accuracy of ML techniques using various missing data imputation techniques.

The outcomes of the DNN algorithm and all the Machine Learning algorithms are also compared. For DNN we considered tuning hyperparameters, comparatively the depth and how many nodes for each layer of the DNN model, to improve the prediction. Although tuning can be helpful as there is no set method for it; based on our required performance, we would have needed to train 3-6 layers with 5-30 nodes. The results show that DNN outperforms the other ML in predicting capability as shown in Table 5 by achieving an F1-Score of 95.00% and an accuracy of 94.32%. As we can also observe in Figs. 6, 7 and 8, the performance of all the ML techniques. Our model predicts stroke using ambiguous information, such as lifestyle and past use of medical services. This will improve diagnosis and lower medical expenses in the future. Additionally, the performance of the DNN approach and all other ML techniques are compared.

**Table 7:** Comparison of the performance between machine learning models that integrate missing data and those that do not.

Aspect	With missing data handling	Without missing data handling
Data size and completeness	Maintains the size of the data by imputing missing values, employing the complete information for training	Rows containing values that are missing are frequently eliminated, hence affecting the amount of sample and information
Bias and Variance	Lowered bias, since imputed data aids in preserving stability and preventing distortion of the parameters of the model	Exclusion of rows or columns may result in significant bias, producing an unrepresentative sample
Impact on Feature Relationships	Imputation maintains inter-feature interactions, resulting in stronger and consistent models	Distorting correlations occur when significant characteristics are missing values, resulting in unreliable predictions
Algorithm Compatibility	Most machine learning methods can be efficiently employed with imputed input	Some approaches (e.g., linear models, neural networks) are incapable of directly accommodating missing values
Computational Efficiency	Imputation methods, such as KNN and MICE, can be highly computational, impacting scalability	Models could show superior computing speed but demonstrate a deficiency in performance stability
Practical Application	Appropriate for sensitive domains (e.g., healthcare) where data integrity is essential for safety	Insufficient for delicate applications; skewed systems may result in significant inaccuracies
Model Interpretability	Models retain interpretability by precise imputation that preserves the structure of the data	Interpretability is compromised by the absence of context and imperfect correlations
Overall Model Performance	Generally superior performance regarding precision, reliability and stability	Unreliable and inconsistent performance resulting from insufficient learning and biases

Table 7 presents a comparison between the performance of machine learning models that handle missing data and those that do not. The findings indicate that the application of missing data imputation techniques maintains dataset size, resulting in enhanced model accuracy. Imputation techniques preserve the interrelationships among features, thereby diminishing bias and variation and facilitating models to produce more reliable and consistent predictions.

However, the absence of missing data management leads to the elimination of rows with absent values, which in turn reduces the size and quality of the dataset. This frequently results in biased outcomes due to a disproportionate sample, reduced feature correlations, and decreased predictive efficacy. Furthermore, models that lack imputation often struggle to generalize to new data sets. While models that eliminate rows may provide enhanced computational speed but display less than optimal stability.

In summary, the table demonstrates that missing data imputation markedly improves model interpretability, performance, and accuracy, particularly in sensitive situations where data completeness is essential. Models employing imputation techniques typically outperform those that merely exclude rows with absent values.

Different existing models have experimented with different datasets. The reason for selecting these models for comparison is that we couldn’t find many works on the CHS dataset. Although different datasets have different sets of attributes, a comparison with these existing models has a wider scope for comparative analysis of our proposed model. The stroke prediction model in^46^ uses the CHS dataset, but the size of the dataset is very small (291 records). Although it achieved an accuracy of 97.7%, the small size of the dataset and the absence of a missing data imputation module are the downsides of the model. From^46^, we observed that if the dataset size is small, then the reported accuracy is high, but the same is not true when the dataset size is large. In^16^, the same thing can be observed for other datasets as well. Irrespective of the attribute selection method (manual or automatic), if the size of the dataset is small (less than 500 records), the reported accuracy is high, and if the dataset is large (more than 1000 records), then the reported accuracy is low.

We acknowledge that relying exclusively on “stroke” and “no disease” records omitted patients with comorbidities, potentially constraining clinical generalizability. This was done to reduce noise and missing data in the CHS dataset, as well as provide a clean baseline for model validation. Although the final dataset is imbalanced and contains a total of 1,840 positive records, extensive data pre-processing, 10-fold cross validation and balanced performance measures reduced bias. Future studies will include multiple conditions and a larger dataset to make it more clinically relevant and reliable.

Although there was no use of post hoc explainable AI methods, to develop an interpretable model, we selected features using IGR with the C4.5 decision tree algorithm, which objectively generated and ranked the most important predictors such as age and hypertension, diabetes, BMI, and smoking status. The ranking provides a transparent and statistically well-supported interpretation of important stroke risk factors, contributing to the clinical understanding of the model outputs. The primary contribution of this work was to establish a flexible preprocessing and classification pipeline for medical data with high missing rate, which the findings demonstrated that IGR could give a reliable balance between interpretability and performance. Finally, further studies will incorporate advanced explanaible AI methods in order to enhance clinical interpretability and drive real-world adoption.

The performance of the preprocessing and imputation pipeline is assessed using the proposed hybrid approach against baseline classifiers in this study. We acknowledge that we would have obtained a more solid benchmark with hybrid and ensemble models. Due to the technological limitations and complexity of the dataset, these distinctions were not compared, at this stage. In our forthcoming work, we looking forward to extending the tests of more advanced hybrid and ensemble models so as to demonstrate that their associativity can be generalized.

*Limitation:* This study has several limitations that although it reached a high prediction accuracy should be mentioned. It lacks external validation so we cannot generalize the proposed model. The experiments were only evaluated on the CHS dataset, and the model has not been tested using external or cross-institutional datasets. Follow-up studies will need to include validation using external and geographically diverse sample populations in order to test the validity of the model and its relevance for clinical application across populations.

Second, the bias of the CHS dataset could be introduced into the dataset. Limitations The data was limited to a specific demographic (older adults from a defined geographic and clinical setting) which may not generalise to wider or more varied populations. The dataset is also intrinsically unbalanced and inconsistent, e.g., there are missing entries, manual recording errors, and unnecessary or irrelevant attributes. These challenges may influence model training and introduce small biases in predictions, which hamper the performance of practical clinical application.

Thirdly, although the hybrid solution effectively addresses missing values problem by KNN imputation, and improves classification performance with DNN, the generalization capability of the model to new or unseen homogeneous/heterogeneous data still remains unknown. Adequate generalization in deep architectures requires large, balanced and representative datasets; however, as described above, despite pre-processing CHS is quite small and heterogeneous. Therefore, it is possible that the current model overfits to patterns specific to this dataset and does not generalize well to changes observed in other populations or health care systems.

Overall, due to strong correlation between features and limited high-quality input data, the model was not able to utilize complex feature interactions. The absence of clinical variables, such as imaging and lab parameters, limits even more the predictive ability of the model. Subsequent multimodal data should be combined, and federated learning frameworks can be used for better robustness, interpretability, and clinical utility by external cohort validation.

The model’s simplicity, requiring 15 routine variables, permits easy integration into an outpatient or primary care setting. It may be a computerized preliminary screening tool in EHR systems for detecting patients at risk for stroke, such that further resource allocation/lifestyle change/tests are performed. This makes the model a practical tool for clinical decision-making and not an alternative to diagnostic evaluation.

Lastly, this work is limited due to the used singular dataset and three-layer DNN model trained with ALO only compared to other optimization methods. This could potentially be addressed in future research by using more complex models, optimizing differently and generalizing better to other datasets. Furthermore, in this paper the proposed hybrid approach is also compared with baseline classifiers in order to investigate the effectiveness of the preprocessing and imputing pipeline. We acknowledge that employing sophisticated hybrid and ensemble models would create a stronger baseline. In addition to technological limitations and the intricacy of the dataset, such comparisons were excluded in this stage. In future research, we intend to broaden the evaluation to include sophisticated hybrid and ensemble frameworks to more thoroughly ascertain the competitiveness and generalizability of the proposed model.

The main contribution of this study lies in the integration and systematic evaluation of established techniques, including feature selection based on the information gain ratio, selective KNN imputation, and deep neural network classification, all within a unified framework for predicting stroke risk using the CHS dataset. The emphasis is on demonstrating the effectiveness and clinical applicability of these integrated methods, rather than introducing a new algorithmic approach..

## Conclusion

This research introduces a stroke prediction model that conducts a semantic analysis of stroke diseases in the elderly employing the CHS dataset. In contrast to prior research, we performed a unique splitting feature importance analysis and a step wise analysis for choosing a restricted set of clinical features. The features selected can effectively identify and predict the likelihood of stroke in the elderly population. Additionally, missing values were imputed in the dataset using KNN imputation algorithm to assess the reliability of the model output. Finally, seven ML algorithms (DNN, RF, SVM, KNN, LR, NB, and DT) were implemented on the selected dataset with a restricted set of clinical features and assessed the overall performance with several evaluation parameters (F1 score, Accuracy, specificity, sensitivity, FPR and precision). The present study introduces an integrated machine learning approach that incorporates feature selection, data imputation and stroke prediction. We extensively compared six state-of-the-art ML techniques with a DNN algorithm and observed that the DNN approaches outperformed all six approaches in predicting stroke with an accuracy of 94.32%. In addition we presented a unique feature selection technique from incomplete dataset by splitting the dataset into two sets: a complete set and an incomplete set and selecting the best feature from the complete set which reduces the complexity of feature selection from dataset with missing values or incomplete dataset. The approach outlined in this work holds significant academic merit as it may precisely forecast the predictive symptoms and beginning of stroke by evaluating fundamental clinical characteristics at a little expense, which might be beneficial for individuals with limited economic resources. In the near future, we will conduct experiments using various combinations of ML models on substantial healthcare datasets. Furthermore, utilizing larger sets of data will enable us to effectively train the DNN model. For our forthcoming endeavor, we plan to collect institutional data and analyze research utilizing diverse outlier detection strategies that warrant further exploration.

Further investigations should extend the present study to compare KNN imputation with state-of-the-art techniques such as MICE, autoencoder-based, or ensemble methods to evaluate their efficacy across various patterns of missing data and may investigate and explore how integrate imputation stage with class imbalance management methods as well as fully automated model tuning algorithms can make models more robust and generalizable. Second, we will compare the proposed IGR–KNN–DNN model with state-of-the-art ensemble learning methods such as XGBoost and CatBoost, and evaluate its generalization performance on an independent external dataset.

**Table 8:** Representation of confusion matrix of all the classifiers employed in the study for comparison. DNN represent the highest true positive and true negative.

ML algorithm	True positive	True negative	False positive	False negative
NN	281	208	18	21
NB	263	193	41	31
LR	278	195	26	29
RF	265	201	34	28
KNN	243	192	45	48
DT	251	191	53	33
SVM	248	189	48	43
**DNN**	**285**	**213**	**12**	**18**

## Data Availability

The dataset is available at https://biolincc.nhlbi.nih.gov/studies/chs/.
